# Exploratory study of brain waves and corresponding brain regions of fatigue on‐call doctors using quantitative electroencephalogram

**DOI:** 10.1002/1348-9585.12121

**Published:** 2020-04-22

**Authors:** Gregory Xavier, Anselm Su Ting, Norsiah Fauzan

**Affiliations:** ^1^ University Malaysia Sarawak Sarawak Malaysia

**Keywords:** doctors, electroencephalogram, fatigue

## Abstract

**Objectives:**

It is common to find doctors working long and odd hours and many at times without rest and sleep. Despite the evidence of adverse risk, jeopardizing patient safety under the hands of fatigue doctors under such working hours has not changed in many places. It has argued that with such training and subsequent experience, such issues with patient safety reduce. Fatigue too is argued as subjective, as those who can withstand the stress still perform. Nevertheless, undeniably working under fatigue is not safe for both the patient and the doctor. This study is a novel attempt to explore and objectify the state of fatigue using quantitative EEG among post‐call doctors.

**Method:**

Seven volunteer post‐call doctors were recruited to go through an EEG recording before and after their on‐call rotation while at rest and subsequently while carrying out Stroop Test, putting their cognitive function at work.

**Results:**

The doctors have worked up to 33 hours in a row and have had sleep of an average of 1.5 hours. It is found that during task there is a statistically significant increase in theta (frontal and occipital regions) and beta (occipital region) band power while at task post‐call. Alpha band power is increased in the frontal and reduced in other regions. Correlation with Stroop Test results indicated that those who have higher alpha, beta, and lower relative theta powers at the frontal region at post‐call rest have higher percentage of correct congruent trials.

**Conclusion:**

The results objectively imply that these fatigue doctors are under more strain while carrying out a task and corresponds to the implicated regions of brain stimulated by the task accordingly.

## 1 INTRODUCTION

Fatigue in normal people is an experience suffered due to prolonged work without due rest. It is a state of extreme tiredness resulting from mental or physical exertion or illness with reduced muscle efficiency and a decrease in response and enthusiasm over prolonged work.^1^ This condition can disappear with adequate rest. However, those with chronic diseases may suffer fatigue as a secondary condition and if it were more than 6 months, it would be diagnosed as Chronic Fatigue Syndrome, otherwise, if of unknown cause a diagnosis of chronic debilitating syndrome would be made.^2^

In the working population, fatigue is a common complaint with approximately 20% reporting it.^1^ The Maastricht Cohort Study carried out in 1998 Netherlands claims prolonged fatigue is common among workers at around 21.9% transecting over 12 sectors and work trades.^3^ The study further reports that there is a prevalence of fatigue among 17.6% healthcare workers.^3^ Other study reports vary from <10% to >40%^1^ and a Taiwan study reports of a 30.9% prevalence.^4^

The medical field is of special concern. This area of work is physical and mentally demanding. Mentally demanding task are ones that need prolonged concentration and focus or constant processing of a multitude of complex task, leading to mental exhaustion.^5^ Doctors directly treat and manage patients and hence subjected as a critical occupation with respect to the fact, if a mistake committed could lead to the death of a patient. Patient safety is compromised when treated by fatigue doctors. The accidents potentially lethal, however, more commonly cause iatrogenic complications and increased morbidity. In addition, fatigue among medical personnel has been linked to reduced professional performance and an increase in medical errors^4^ and also self‐accidents and near misses of injuries. Locally in Malaysia, most of the reported motor vehicle accidents suffered by doctors were attributed to on‐call fatigue due to extended working hours and sleep deprivation.^6^ There is also further inclusion of job demands and expectations.^7^ Prolonged fatigue can further create a vicious cycle of affective changes which can lead and also cause depression, anxiety, confusion, and anger.^8^ Changes in cognitive performances are also reported and that could also be the cause of an effect of lowered psychomotor performances.^9^ Hence, possibly these have produced and contributed to the toxic work environment commonly spoken off in hospitals.

Many studies have used the electroencephalogram (EEG) to study fatigue. It is an instrument to detect bioelectrical brain waves (extracellular current flow). EEG signals are indicated to be very predictive and reliable tool to detect alertness levels^10^ and could be used in the prevention of fatigue or as a trigger in counter‐measure instruments.^11^ At fatigue, there is a decrease in physiological arousal, slowed sensorimotor functions, and impaired information processing, impairing the workers’ ability to respond effectively in emergencies or unusual situations.^12^ These changes can be deduced by analyzing the four types of brain waves detected by EEG. They are Delta (±0 to 4 Hz), theta (4‐8 Hz), alpha (8‐13 Hz), and beta (13‐20 Hz).^13^ Delta waves are more frequent during sleep. An early stage of drowsiness is indicated by an increase in theta waves. Alpha waves reflects a state of relaxed wakefulness which decrease with concentration, stimulation, or visual fixation,^14^ a state where the worker is fatigued enough to fall asleep.^13^ Beta waves are increased while alert and decreases during drowsiness.^15^

The EEG would be able to explain the brain electrophysiological state of a fatigue doctor's mind while at work. The objective of the study is to explore the electrophysiological state of doctors before and after the on‐call rotation, while at rest and conducting a task. We aim to identify any presence of apparent brain wave changes and its relation to brain regions. By understanding the state of mind of fatigue doctors objectively using an EEG, cautions their need to continue to work but in need of rest. This study of using EEG to detect fatigue in doctors is novel. Most studies conducted were on motor‐vehicle operators under simulated driving or piloting for the development of counter‐measure instruments for the prevention of accidents during fatigue.

## 2 METHODOLOGY

### 2.1 Study subjects

**FIGURE 1:**
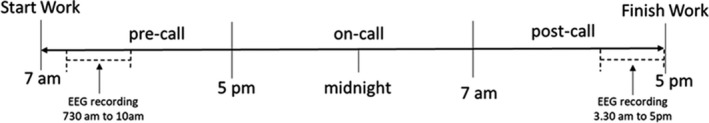
On‐call rotation work schedule

This study includes doctors who work under on‐call rotations from a tertiary hospital. The volunteered subjects were required to undergo two EEG recordings. One recording is before they go to work and the other after they finish their on‐call day at post‐call. The on‐call rotation schedule is as presented in Figure 1. At their post‐call, they answered the Fatigue Assessment Scale (FAS) questionnaire to ascertain their fatigue state of mind subjectively. The FAS is a 10‐item, and is unidimensional consisting of a five‐point rating scale (1‐5). The subject would rate each item according to how they feel the last 24 hours and finally the total scores would be added up. A score of 10‐21 is considered having no fatigue. A score of 22‐50 indicates there is fatigue.^16^, ^17^ Doctors who have any kind of neurological, including mental illnesses were excluded.

### 2.2 Experimental protocol

**FIGURE 2:**
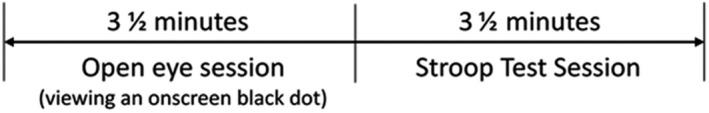
EEG recording protocol

The doctors were subjected to a 3.5 minute open eye EEG recording session followed by another 3.5 minute session of Stroop Test while EEG recording continued.^18^, ^19^ This procedure provides different effects on physiological responding and behavior of the subjects’ brain waves at “arousal” and “rest”^19^, ^20^ and enough recording data to calculate the magnitude of the waves in study.^21^ The EEG recording sessions is as presented in Figure 2. During the eye open session, the subject is required to look at an on‐screen black dot.

The Stroop Test, tests the susceptibility to interference in information processing of a subject.^22^, ^23^ The subjects had to identify the congruent and incongruent color of the words displayed, correctly and as fast as possible. The test results are recorded in milliseconds. The correct percentage, number of trials attempted and the average speed taken to answer the test parameters are recorded. The entire recording session took 7 minutes only (Figure 2).

### 2.3 EEG recording and processing

We used the MITSAR cap EEG (post‐call) and Nicollete EEG instruments (pre‐call) and amplifiers, arranged following the 10‐20 International system for recording the brain waves. WinEEG software is used to measure the absolute power of those waves (microvolt, µV^2^) after removal of its artifacts. Recording was done using the Average Reference Montage, at a sampling rate of 1 kHz, low pass filter of 0.3 Hz and a high pass filter of 50 Hz and notch setting at 45‐55 Hz. We categorized and analyzed the brain waves following the electrode placements according to regions^24^, ^25^: frontal region includes Fz, Fp1, Fp2, F3, F4, F7, F8. Centro‐parietal: Cz, Pz, C3, C4, P3, P4. Temporal: T3, T4, T5, T6, and Occipital: O1 and O2.

The brain wave frequencies of alpha, beta, and theta is converted into magnitude as microvolt (µV^2^) power^26^ using the software. Two fatigue indexes adapted for analysis are the (*α* + *θ*)/*β* and *α*/*β* indexes.^27^, ^28^, ^29^ We study the relative power of these frequencies besides their absolute powers.

### 2.4 Statistical analysis

The analysis of brain waves follows the specified brain regions to observe their respective prominence, in addition to analyzing the observation of brain wave changes between pre‐call and post‐call, at rest and while at task. The Wilcoxon Signed Ranks test is used to ascertain any significance in those observed changes. The results of the Stroop Test are correlated to observe its relationship with the brain wave changes.

## 3 RESULTS

Seven post‐call doctors volunteered for the study including two females and five males with a mean (SD) age of 30.1 (1.57) years. Most of them were from the surgical department with a mean (SD) working experience of 5.14 (1.77) years. The mean (SD) duration of sleep they had were 1.5 (0.87) hours, being awake for a mean (SD) 33.3 (1.98) hours. They have all denied consuming alcohol, tea, and smoking the past 24 hours. Only two consumed coffee during their on‐call. Using the FAS questionnaire all the subjects scored 22 marks and above, subjectively expressing they are in the state of fatigue at post‐call.

During the 3.5 minutes of Stroop Test task, there was a significant difference in the speed of answering the trials. Congruent trials were answered significantly faster compared to incongruent trials for both pre‐call (*Z* = −2.371, *P* < .05) and post‐call (*Z* = −2.371, *P* < .05) but the correct percentage correct was not significant. Nevertheless, more mistakes are seen despite faster attempt speed at post‐call for the congruent trials (post‐call). The number of trials attempted were, however, significantly lesser pre‐call with a mean (SD) of 107 (12), *Z* = −2.201, *P* < 005. The mean (SD) number of trials attempted post‐call were 125(6.2).

Correlation analysis found that at rest, post‐call, those with higher alpha (*r* = .757, *P* = .049) and beta power (*r* = .809, *P* = .028) at the frontal region, and higher beta (*r* = .802, *P* = .03) and relative alpha power (*r* = .757, *P* = .049) globally have higher percentage of correct congruent trials. This also coincides with the findings of lower relative theta at the frontal (*r* = −.802, *P* = .049) and occipital (*r* = −.757, *P* = .049) regions. Other correlation findings were weak and inclusive due to limitations.

Absolute theta waves increased while conducting the task in all brain regions. Significant increases were seen at pre‐call (global, frontal, and centro‐parietal) but only at frontal and occipital at post‐call. This pattern is also seen similar both at pre‐ and post‐call. Relative theta power also increased at task at both pre‐ and post‐call, however, its magnitude is larger globally at post‐call be it at rest or while at task (Table 2). Theta wave prominence is higher in all regions while at task for both pre‐ and post‐call. At rest, post‐call theta power is generally lower than that of pre‐call except at the frontal region. At task, theta power actually decreases between pre‐ and post‐call except at the temporal region but its relative magnitude is higher at post‐call.

Absolute alpha power shows increase while at task, pre‐call in all brain regions but reduced likewise at post‐call (Table 1). The changes were not otherwise significant. Its relative power had all decreased pre‐ and post‐call but the decrease was more significant post‐call at global, temporal, and occipital regions. The relative magnitude of alpha was also higher pre‐call at rest and task (Table 2). Alpha wave prominence is higher in all regions except frontal at rest, both for pre and post‐call. Theta is otherwise prominent in that region. The absolute and relative alpha power is lower at task during post‐call compared to pre‐call. At rest, similar pattern is observed but there is an increase instead seen globally at the centro‐parietal region.

**TABLE 1:** Absolute brain wave power in microvolt (µV^2^)

	Theta						Alpha						Beta					
	Rest			Task			Rest			Task			Rest			Task		
	Pre‐call	Post‐call	*P*‐value	Pre‐call	Post‐call	*P*‐value	Pre‐call	Post‐call	*P*‐value	Pre‐call	Post‐call	*P*‐value	Pre‐call	Post‐call	*P*‐value	Pre‐call	Post‐call	*P*‐value
Global	2.408	2.404	.735	4.928	4.960	.866	3.053	3.440	.612	3.743	2.966	1.000	1.337	1.174	1.000	2.915	3.077	.866
frontal	1.208	1.329	.612	3.082	2.968	1.000	1.191	1.152	1.000	1.745	1.361	.237	0.293	0.609	.176	0.806	1.228	.866
Centro‐parietal	0.587	0.365	.735	1.735	0.448	.499	0.827	1.006	.612	1.641	0.491	.612	0.210	0.229	.735	0.795	0.785	.612
temporal	0.662	0.535	.237	1.058	1.087	.866	0.952	0.892	.612	1.367	0.745	.866	0.476	0.270	.499	0.760	0.717	.866
occipital	0.316	0.174	.237	0.632	0.458	.866	0.493	0.389	.735	0.593	0.369	.866	0.118	0.066	.866	0.245	0.348	.612

**TABLE 2:** Relative brain wave power in microvolt (µV^2^)

	Relative theta						Relative alpha							Relative beta					
	Rest			Task			Rest			Task				Rest			Task		
	Pre‐call	Post‐call	*P*‐value	Pre‐call	Post‐call	*P*‐value	Pre‐call	Post‐call	*P*‐value	Pre‐call	Post‐call	*P*‐value	Pre‐call		Post‐call	*P*‐value	Pre‐call	Post‐call	*P*‐value
Global	0.341	0.377	.866	0.456	0.479	.499	0.448	0.436	1.000	0.362	0.292	.180	0.211		0.187	.735	0.181	0.230	.176
Frontal	0.448	0.440	.735	0.531	0.551	.612	0.425	0.372	.612	0.310	0.258	.063	0.127		0.188	.091	0.159	0.192	.398
Centro‐parietal	0.336	0.321	.866	0.404	0.354	.310	0.489	0.505	.866	0.405	0.377	.091	0.175		0.174	.499	0.192	0.269	.128
temporal	0.240	0.349	.176	0.321	0.439	.128	0.509	0.466	.310	0.453	0.308	.02*	0.251		0.185	.499	0.226	0.252	1.000
occipital	0.248	0.333	.128	0.332	0.411	.237	0.553	0.533	.866	0.441	0.338	.043*	0.198		0.134	.128	0.227	0.251	.735

* Significant *P* < .05.

Absolute beta showed global increase pre and post‐call at task. Significant changes seen at pre‐call globally (*Z* = −2.197, *P* = .028), centro‐parietal (*Z* = −2.028, *P* = .048), and occipital (*Z* = −2.366, *P* = .018) (Table 1). Post‐call significant change was only seen in the occipital region. (*Z* = −2.028, *P* = .043). Relative beta at post‐call rest is much lower than pre‐call rest and higher for task. At pre‐call, relative beta increased in all regions except globally and temporal at task (Table 2). The magnitude of beta power at rest is generally lower at post‐call but when at task its magnitude is increased and even higher than pre‐call at task.

(*α* + *θ*)/*β* index had a significant decrease with *Z* = −2.028, *P* = .043 in the occipital region. The centro‐parietal and temporal regions also showed decrease. Globally and at the frontal region, during post‐call task (*α* + *θ*)/*β* there is increase in power. Magnitude of the (*α* + *θ*)/*β* index was lower at rest and pre‐call than post‐call except at temporal and occipital region. The magnitude increased globally and in the frontal and temporal regions when at task post‐call.

The *α*/*β* index had generally lower magnitude during post‐call task compared to pre‐call task but at rest, it is only lower at the frontal and temporal region. Otherwise, a general decrease is seen during task pre‐call (Table 3). At the occipital region post‐call the *α*/*β* index had a significant decrease with *Z* = −2.197, *P* = .028 during task. Similar decrease is also seen except at the frontal region.

**TABLE 3:** Brain wave algorithmic power in microvolt (µV^2^)

	*α*/*β*						(*α* + *θ*)/*β*					
	Rest			Task			Rest			Task		
	Pre‐call	Post‐call	*P*‐value	Pre‐call	Post‐call	*P*‐value	Pre‐call	Post‐call	*P*‐value	Pre‐call	Post‐call	*P*‐value
Global	3.547	3.589	1.000	2.483	2.237	.310	5.850	5.969	.866	5.726	6.287	.310
Frontal	3.844	2.829	.176	3.236	3.098	.398	6.295	5.876	.499	8.073	11.333	.735
Centro‐parietal	4.317	4.346	.866	2.829	2.342	.128	6.591	6.438	.237	5.500	4.475	.612
Temporal	3.557	4.171	.612	2.307	1.971	.612	5.058	6.703	.128	3.965	4.938	.398
Occipital	4.351	5.346	.398	2.542	1.894	.310	5.808	7.943	.128	4.376	4.303	1.000

At pre‐call in general the changes between rest and task are almost similar. The consistent difference noted between pre‐call and post‐call during task is with absolute alpha power. Absolute alpha power decreases post‐call but is found to be increased pre‐call in all brain regions. At pre‐call the prominence of waves are also similar between work and task but during pre‐call theta is also prominent at the centro‐parietal region. The power values between pre‐call and post‐call of rest and task showed no significant differences throughout.

## 4 DISCUSSION

The working hours for on‐call doctors here are typically from 7am to 5pm the next day, overnight (Figure 1). This is 33 hours of work in a row. In a government, tertiary hospital located in the city the workload is high, hence, there is lack of rest or sleep. The average sleep obtained by the doctors in this study is 1.5 hours. In general, 17‐19 hours of without sleep is the upper limit for safely performing safety‐sensitive work, after which the performance would be equivalent to 0.05% Blood Alcohol Concentration (BAC) and 0.1% BAC if the awake time was between 18 and 20 hours. The limit for Driving Under Influence (DUI) (under alcohol) in Australia is 0.08% BAC; equivalent to around 14‐15 hours without sleep.^30^ In other words, the subject's state of mind when not having enough sleep can be equated to a person who is under alcohol influence. This also justifies that on‐call doctors would have a significant form of fatigue mentally and physically even if they subjectively do not feel so.

The frontal region of the brain, functions as a center for personality, behavior, emotions, judgment, planning, problem solving, body movement (motor strip) intelligence, concentration, and self‐awareness. The parietal covering the central regions functions as a center for interpreting language and words and the site for the sensory and motor strip. The occipital region deals mainly with the interpretation of visual stimuli and the temporal region deal more with hearing sequencing and organization.^31^ All these functions are relayed and processed by five different brain waves: alpha, beta, theta, delta, and gamma. Delta and gammas waves are not studied here due to their less significance in fatigue development and usually studied during sleep.

During fatigue, there is a decrease in physiological arousal, slowed sensorimotor functions, and impaired information processing, impairing the ability to respond effectively in emergencies or unusual situations.^12^ An increase in theta waves indicates an early stage of drowsiness. Alpha waves reflect a relaxed wakefulness state and decrease with concentration, stimulation, or visual fixation.^14^ The person is fatigued enough to fall asleep when he/she has more of these slow waves.^13^ In short, the waves that can indicate fatigue are when there is reduced beta wave (the waking consciousness and reasoning) and increase in theta waves (the light meditation and sleeping). Alpha wave changes but is dependent on the type of work done. If the work requires active mental concentration, we see a reduction. However, if it was more of a monotonous task it would increase.^26^, ^32^, ^33^ In this study, we see that absolute alpha waves are most prominent pre‐ and post‐call at rest and theta waves at work. However, these alpha waves reduce in magnitude at post‐call as compared to increase during pre‐call. These leave more theta and beta waves being emitted in all regions indicating that the subject is in the state of fatigue. The state of drowsiness caused by the evident lack of sleep (theta waves) and the lack of mind relaxation (alpha waves) with high brain activity (beta waves), causes strain and stress and lack of focus.

The Stroop Test assesses the ability of the brain to inhibit cognitive interference in a stimulus.^22^ Therefore, we expect that the frontal and occipital regions to show more beta wave prominence rather than at the parietal but instead theta waves superseded with reduced alpha waves during post‐call. Increase of alpha wave is instead seen with increased theta wave pre‐call. The frontal region determines the inhibitive ability, whereas the occipital region processes the visual stimulus. The parietal processes more of motor (stroking of fingers on keypad) and other sensory stimuli not tested with a Stroop Test. However, the results of this study concur with the objective of a Stroop Test, whereby despite faster attempts on trials, the congruent trials had more mistakes. On the contrary, incongruent trials fared better post‐call. This could be due to the level of education of doctors and their trained experience to remain focused despite being fatigue, in addition to other confounding factors such as mood, emotions, and boredom. Furthermore, the doctors were recruited while they were working, hence, there could exist some bias.

When given a task, we see that theta waves instead gained more prominence in all regions except the centro‐parietal region. Here the beta wave is prominent however; the relative theta and relative alpha are higher than relative beta powers. Higher beta power is expected in this region^31^ of functional motor processing but, we find a low relative beta power instead. In addition, absolute alpha decreases post‐call as opposed to increasing pre‐call during task (Table 1). This implies the subject is nevertheless in the state of fatigue even in this region.

The study of fatigue among drivers (under simulation) displayed an increase in theta. Alpha, and beta waves.^26^, ^32^, ^33^ It is understood and as expected a monotonous work will tend to induce relaxation with reduced attention and when prolonged, will indefinitely change to sleepiness.^33^ In this study, we find that the theta and beta waves increased as the previous studies however, the alpha wave is increased in the frontal region and reduced in all other regions post‐call while conducting task. However, the findings from drivers are similar to the pre‐call doctors while carrying out task.

The Stroop Test increases the cortical arousal for visual processing, hence reducing the alpha waves in all regions except at the frontal region and increase in beta wave for all regions at post‐call. Since the frontal region is the center for decision‐making, the persistence of alpha wave could imply better congruent results at pre‐call but lowered alpha post‐call and that corresponded with more mistakes. The increase of beta waves in stimulatory task reduces the fatigue algorithm powers, hence it differs with studies conducted with monotonous task which produce an increase.^32^ The difference in a given task produces different brain wave changes especially seen with the alpha wave. Monotonous task increases alpha but stimulatory task reduces it. If a fatigue person's theta is persistent, the compensatory need to remain alert increases the beta power coinciding with reduced alpha power.

This study has particularly seen a more statistically significant result in the occipital region. This display of results reflects that the Stroop Test, which is a visually stimulating mind task, did show significantly the increase in absolute beta and the corresponding reduction of algorithm wave power. In tandem, there is a significant reduction of relative alpha power and a significant raise in absolute theta power. In addition to an increase in absolute alpha, post‐call is contrary to its decrease during pre‐call. This further reflects that the subjects are indeed more fatigue when given a mind stimulating task (Table 2) and that replicates the tasks of doctors.

When assessing fatigue, using EEG, we would need to consider the nature of the job. The doctors’ profession is a job, which requires high level of thinking capacity, which includes judgment, planning, problem solving, decision‐making, concentration, and a degree of intelligence level. Long working duration, puts stress on the mind and from the results obtained in this study the persistent level of theta and its significant increase while given a task reflects the stress of mental loading. Increases in beta power and reduced alpha power reflect mental loading. The increase in alpha wave at the frontal region could reflect the compensatory reaction to remain at focus while at work. This could be why at task pre‐call alpha power has higher magnitude globally compared to when at post‐call. In other regions, beta wave power compensates the decrease of alpha power, reflecting the strain needed to focus. During the duration of the EEG recording and time frame of the task, this study reflects that the fatigue mind takes a longer duration to process incongruent trials with lesser, total correct trials.

## 5 LIMITATIONS

Despite this study showing promising results, the sample size is, however, small to magnify for statistical significance and consistency in all the results. The study conducted, was at the hospital neurology clinic and during working hours. This caused logistic limitations and the need of two EEG machines. Nevertheless, brain wave sampling from both instruments is the same following manufacturing specifications that follow standards. Furthermore, the situation hampered recruitment in a time‐limited study. As much as this environment can best elicit the doctors’ state of mind via EEG objectively, the cognitive test results are subject to their mood state. Hence, any correlations are nevertheless guarded.

## 6 CONCLUSION

In conclusion, the prominence of theta wave power with reduced alpha and increased beta power objectively identifies the fatigue state of mind of doctors. Stimulatory task decreases alpha wave power and increases beta wave power when fatigue is seen during post‐call.

## DISCLOSURE

*Approval of the research protocol*: The study is registered with the National Medical Research Registry and ethical approval obtained from its Medical Research Ethics Committee (MREC) with the number NMRR‐18‐3104‐44693. *Informed consent*: Informed consent was obtained from all the respondents. The Malaysian Brain, Mind and Neuroscience Research Foundation has provided funds for this study. *Registry and registration no. of the study/trial*: N/A. *Animal studies*: No animals were recruited for the study. *Conflict of interest*: The authors declare no conflict of interest in conducting the study. We acknowledge the Director General of Health Malaysia for allowing the use of the Ministry of Health facilities and staff. We also thank University Malaysia Sarawak for their support and use of their resources.

## AUTHOR CONTRIBUTION

Gregory Xavier: data collection and writing; Anselm Su Ting: conceived idea, supervising, writing; Norsiah Fauzan: supervising, data collection.

## References

[1] Bültmann U , Kant I , Kasl SV , Beurskens AJHM , van den Brandt PA . Fatigue and psychological distress in the working population. J Psychosom Res. 2002;52(6):445‐452. 12069868 10.1016/s0022-3999(01)00228-8

[2] Bates DW , Schmitt W , Buchwald D , et al. Prevalence of fatigue and chronic fatigue syndrome in a primary care practice. Arch Intern Med. 1993;153(24):2759‐2765. 8257251

[3] Kant I . An epidemiological approach to study fatigue in the working population: the Maastricht Cohort Study. Occup Environ Med. 2003;60(>90001):32i‐39. 10.1136/oem.60.suppl_1.i32 PMC1765733 12782745

[4] Ho J‐C , Lee M‐B , Chen R‐Y , et al. Work‐related fatigue among medical personnel in Taiwan. J Formos Med Assoc. 2013;112(10):608‐615. 23809098 10.1016/j.jfma.2013.05.009

[5] van der Linden D , Frese M , Meijman TF . Mental fatigue and the control of cognitive processes: effects on perseveration and planning. Acta Psychol (Amst). 2003;113(1):45‐65. 12679043 10.1016/s0001-6918(02)00150-6

[6] Barger LK , Cade BE , Ayas NT , et al. Extended work shifts and the risk of motor vehicle crashes among interns. N Engl J Med. 2005;352(2):125‐134. 15647575 10.1056/NEJMoa041401

[7] Sluiter JK . Need for recovery from work related fatigue and its role in the development and prediction of subjective health complaints. Occup Environ Med. 2003;60(>90001):62i‐70. 10.1136/oem.60.suppl_1.i62 PMC1765724 12782749

[8] Gaba DM , Howard SK . Fatigue among clinicians and the safety of patients. N Engl J Med. 2002;347(16):1249‐1255. 12393823 10.1056/NEJMsa020846

[9] Orton DI , Gruzelier JH . Adverse changes in mood and cognitive performance of house officers after night duty. BMJ. 1989;298(6665):21‐23. 2492842 10.1136/bmj.298.6665.21 PMC1835335

[10] Lal SKL , Craig A , Boord P , Kirkup L , Nguyen H . Development of an algorithm for an EEG‐based driver fatigue countermeasure. J Safety Res. 2003;34(3):321‐328. 12963079 10.1016/s0022-4375(03)00027-6

[11] Huang K‐C , Huang T‐Y , Chuang C‐H , et al. An EEG‐based fatigue detection and mitigation system. Int J Neural Syst. 2016;26(04):1650018. 27121994 10.1142/S0129065716500180

[12] Mascord DJ , Heath RA . Behavioral and physiological indices of fatigue in a visual tracking task. J Safety Res. 1992;23(1):19‐25.

[13] Akerstedt T , Gillberg M . Subjective and objective sleepiness in the active individual. Int J Neurosci. 1990;52(1‐2):29‐37. 2265922 10.3109/00207459008994241

[14] Stern JM , Engel J , Ovid Technologies I . Atlas of EEG Patterns. Philadelphia, PA: Lippincott Williams & Wilkins; 2005 https://trove.nla.gov.au/version/13069668. Accessed August 16, 2019.

[15] Hong J , Eoh MKC , Electroencephalographic study of drowsiness in simulated driving with sleep deprivation; 2005 https://www.researchgate.net/publication/223394934_Electroencephalographic_study_of_drowsiness_in_simulated_driving_with_sleep_deprivation. Accessed October 5, 2018.

[16] Vries JD , Michielsen HJ , Heck GLV . Assessment of fatigue among working people: a comparison of six questionnaires. Occup Environ Med. 2003;60(suppl 1):i10‐i15. 12782741 10.1136/oem.60.suppl_1.i10 PMC1765720

[17] Michielsen HJ , De Vries J , Van Heck GL . Psychometric qualities of a brief self‐rated fatigue measure: the Fatigue Assessment Scale. J Psychosom Res. 2003;54(4):345‐352. 12670612 10.1016/s0022-3999(02)00392-6

[18] Mecarelli O . Clinical Electroencephalography. Rome: Springer; 2019 p. 809.

[19] Barry RJ , Clarke AR , Johnstone SJ , Magee CA , Rushby JA . EEG differences between eyes‐closed and eyes‐open resting conditions. Clin Neurophysiol. 2007;118(12):2765‐2773. 17911042 10.1016/j.clinph.2007.07.028

[20] Barwick F , Arnett P , Slobounov S . EEG correlates of fatigue during administration of a neuropsychological test battery. Clin Neurophysiol. 2012;123(2):278‐284. 21798799 10.1016/j.clinph.2011.06.027 PMC3206198

[21] Kropotov JD , Quantitative EEG . Event‐Related Potentials and Neurotherapy. 1st ed Moscow: Academic Press.

[22] Scarpina F , Tagini S . The Stroop Color and Word Test. Front Psychol. 2017;8:557 10.3389/fpsyg.2017.00557 28446889 PMC5388755

[23] Jensen AR , Rohwer WD . The Stroop color‐word test: a review. Acta Psychol (Amst). 1966;25(1):36‐93. 5328883 10.1016/0001-6918(66)90004-7

[24] Ferreira C , Deslandes A , Moraes H , et al. Electroencephalographic changes after one nigth of sleep deprivation. Arq Neuropsiquiatr. 2006;64(2B):388‐393. 16917606 10.1590/s0004-282x2006000300007

[25] Fan X , Zhou Q , Liu Z , Xie F . Electroencephalogram assessment of mental fatigue in visual search. Biomed Mater Eng. 2015;26(Suppl 1):S1455‐1463. 26405908 10.3233/BME-151444

[26] Lal SKL , Craig A . Driver fatigue: electroencephalography and psychological assessment. Psychophysiology. 2002;39(3):313‐321. 12212650 10.1017/s0048577201393095

[27] Jap BT , Lal S , Fischer P , Bekiaris E . Using EEG spectral components to assess algorithms for detecting fatigue. Expert Syst Appl. 2009;36(2):2352‐2359.

[28] Eoh HJ , Chung MK , Kim S‐H . Electroencephalographic study of drowsiness in simulated driving with sleep deprivation. Int J Ind Ergon. 2005;35(4):307‐320.

[29] Cheng S , Lee H , Shu C , Hsu H . Electroencephalographic study of mental fatigue in visual display terminal task. J Med Biol Eng. 2007;27(3):124‐131.

[30] Williamson A , Feyer A‐M , Friswell R , Finlay‐Brown S . Development of Measures of Fatigue: Using an Alcohol Comparison to Validate the Effects of Fatigue on Performance. 2000;79. 10.1016/s0001-4575(00)00045-2 11235793

[31] Sherwood L . Human Physiology. 6th ed Donnybrook: Thomson Brooks; 2007 p. 275.

[32] Ma J , Gu J , Jia H , Yao Z , Chang R . The relationship between drivers’ cognitive fatigue and speed variability during monotonous daytime driving. Front Psychol. 2018;9:459. 29670563 10.3389/fpsyg.2018.00459 PMC5893797

[33] Papadelis C , Kourtidou‐Papadeli C , Bamidis PD , et al. Indicators of sleepiness in an ambulatory EEG study of night driving. Conf Proc Annu Int Conf IEEE Eng Med Biol Soc IEEE Eng Med Biol Soc Annu Conf. 2006;1:6201‐6204. 10.1109/IEMBS.2006.259614 17946748

